# Supplementary material for: Distinct roles of dentate gyrus and medial entorhinal cortex inputs for phase precession and temporal correlations in the hippocampal CA3 area
Source: Nat Commun. 2025 Jan 2;16:13. doi: 10.1038/s41467-024-54943-2 (PMC11696047; doi:10.1038/s41467-024-54943-2)
Supplement: Supplementary file 1 — Supplementary Information [file 41467_2024_54943_MOESM1_ESM.pdf]

Figure S1

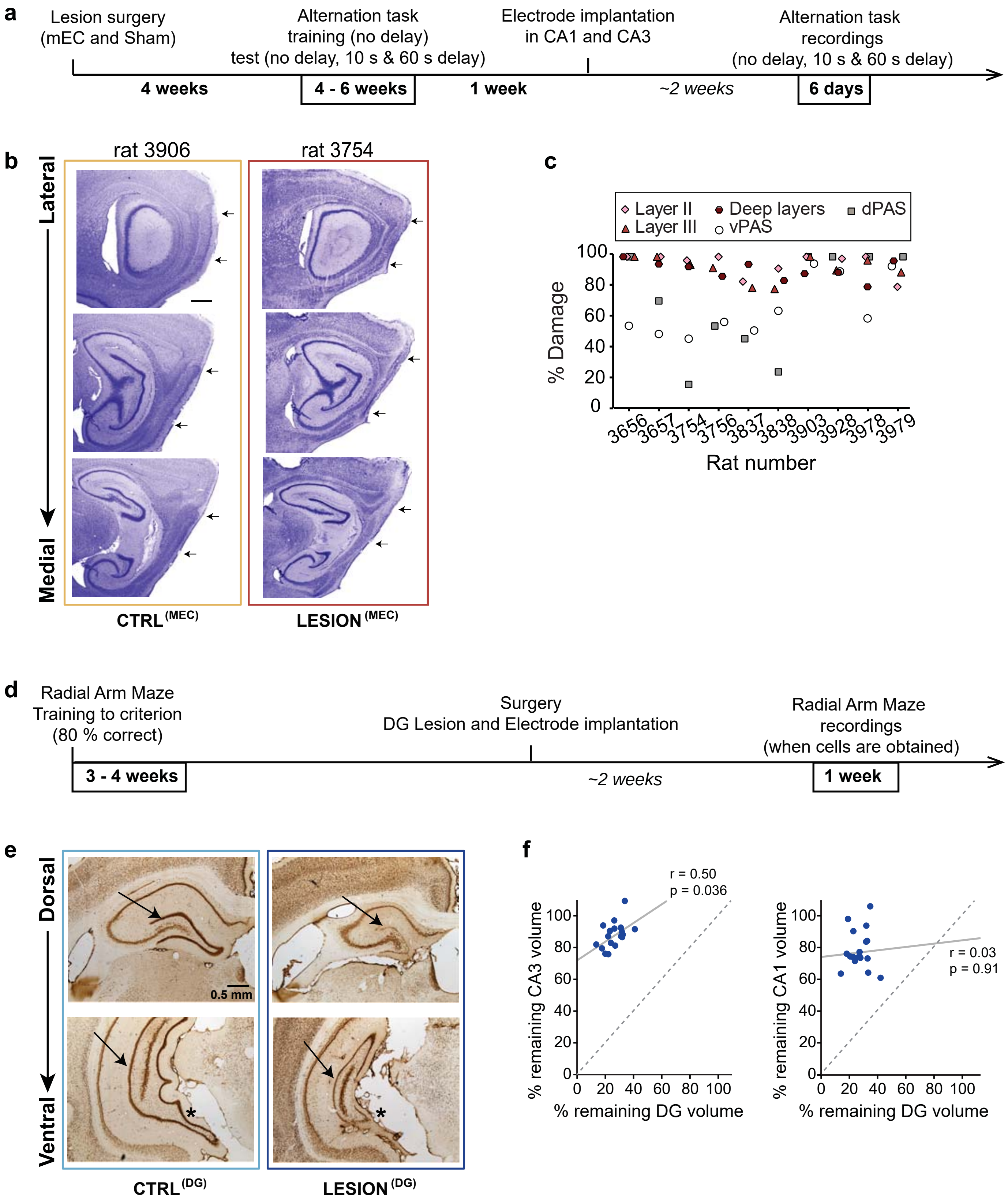
a
Lesion surgery (mEC and Sham)
Alternation task training (no delay) test (no delay, 10 s & 60 s delay)
Electrode implantation in CA1 and CA3
Alternation task recordings (no delay, 10 s & 60 s delay)
4 weeks
4 - 6 weeks
1 week
~2 weeks
6 days
b
rat 3906
rat 3754
Lateral
Medial
CTRL (MEC)
LESION (MEC)
c
Layer II
Deep layers
dPAS
Layer III
vPAS
% Damage
100
80
60
40
20
0
3656 3657 3754 3756 3837 3838 3903 3928 3978 3979
Rat number
d
Radial Arm Maze Training to criterion (80 % correct)
Surgery DG Lesion and Electrode implantation
Radial Arm Maze recordings (when cells are obtained)
3 - 4 weeks
~2 weeks
1 week
e
Dorsal
Ventral
0.5 mm
CTRL (DG)
LESION (DG)
f
r = 0.50
p = 0.036
% remaining CA3 volume
% remaining DG volume
r = 0.03
p = 0.91
% remaining CA1 volume
% remaining DG volume
0 20 40 60 80 100

Figure S1. Quantification of DG and MEC lesions. Experimental timelines and quantification of lesion size are adapted from previous publications with permission (MEC lesions: from Fig. 1a, 1d, S1c in ref. 55; DG lesions: from Fig. 1c, S1d in ref. 12). **a**, Experimental timeline for MEC lesion rats and their controls. MEC lesion or sham surgeries (control) were performed prior to training on the spatial alternation working memory task. **b**, Series of sagittal sections from a control (left) and MEC-lesion (right) rat. Horizontal arrows indicate the dorsal and ventral borders of MEC. Scale bar, 500 µm. **c**, Percentage of MEC lesioned in each animal. Layers of the MEC (layer II, layer III, deep layers V/VI) and adjacent regions (dorsal parasubiculum, dPAS; ventral parasubiculum, vPAS) are distinguished by unique symbols. In the MEC lesion group (n = 10), the Cavalieri method was used to quantify the lesion extent. A total of 93.0% of MEC was completely lesioned (95.3% of layer II, 92.4% of layer III, and 91.4% of deep layers), and the majority of tissue was spared in adjacent regions. **d**, Experimental timeline for DG lesion rats and matched controls. DG lesion or sham surgeries (control) were performed after training on the spatial working memory task. Rats were trained to criterion (80% correct choice) prior to surgery. **e**, Coronal brain sections from the dorsal and ventral hippocampus, labeled with the neuronal marker NeuN, of a control (left) and DG-lesion (right) rat (at the same coordinates from bregma). Arrows point to the dentate granule cell layer. Asterisks highlight the reduction in ventral DG volume. Scale bar, 0.5 mm. **f**, Percent total volume of the CA3 (left) and CA1 (right) cell layers in comparison to the DG cell layer in each DG-lesion animal. Volume was quantified using the Cavalieri method. Damage to CA3 and CA1 was minimal, while loss of the DG granule cell layer was extensive after injection of colchicine, a drug that is selectively toxic for DG granule neurons.

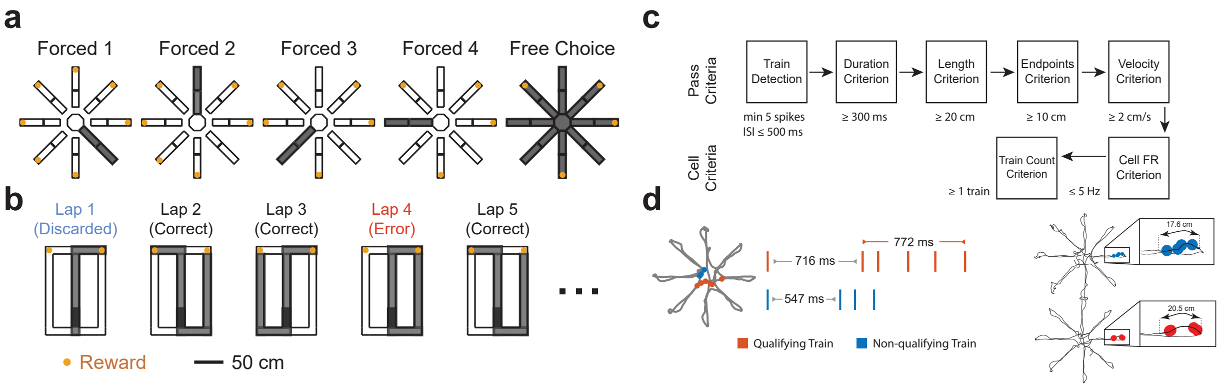

Figure S2. Behavioral tasks and spike train detection. a, In the spatial working memory task on the 8-arm radial maze, rats retrieved food from the end of each arm during each trial. The first four arms were chosen in pseudorandom order by the experimenter (Forced 1, Forced 2, Forced 3, Forced 4), and rats then had to collect the food from the remaining four arms (Free Choice). The gray shading indicates accessible arms. **b**, In the delayed alternation task on the figure-8 maze, rats were allowed to choose freely between two reward locations on the first lap and then had to alternate between reward locations to receive rewards on subsequent laps after briefly waiting in a delay zone between choices. An example sequence of laps is shown with gray shading indicating the maze regions covered on each lap. The tasks on the radial 8-arm maze task and on the figure-8 maze are both hippocampus-dependent spatial working memory tasks [12, 93]. **c**, Spike trains were detected and a series of criteria were then applied to ensure that trains covered a sufficient distance and duration. In addition, cell selection criteria were used to exclude interneurons and to include only cells with a minimum of at least one train. **d**, Examples that qualified or did not qualify as trains. Left, for a CTRL$^{(DG)}$ cell, two spike trains and their corresponding spatial locations (red circles, spikes in qualifying trains; blue circles, spikes that did not qualify for trains due to insufficient number of spikes in train). Right, two trains that qualify based on train detection criteria, but the top example is excluded because the path length during the train is < 20 cm.

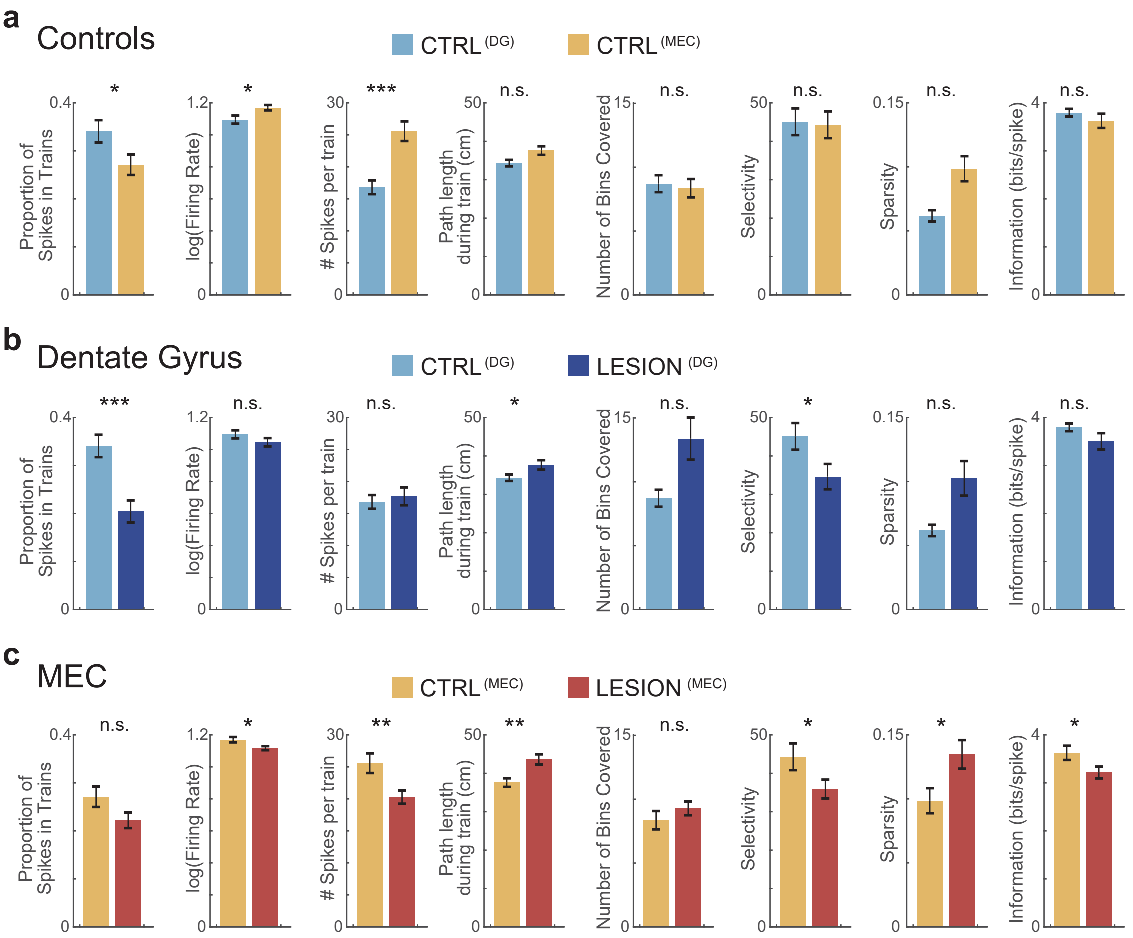

Figure S3. Characteristics of CA3 spike trains showed only minor differences across the two spatial working memory tasks and between control and lesion rats. a, Comparisons between control data in the 8-arm radial maze (light blue) and the figure-8 maze (yellow). Data are from periods of mobility (≥2 cm/s) and consist of trains obtained from *n* = 97 cells in CONTROL$^{(DG)}$, *n* = 68 cells in LESION$^{(DG)}$, *n* = 118 cells in CONTROL$^{(MEC)}$, and *n* = 169 cells in LESION$^{(MEC)}$. Spike trains of control CA3 cells (see Methods for criteria) showed only minor differences across the two spatial working memory tasks in the proportion of spikes assigned to trains (z-statistic = 2.55, $p = 0.0108$), in-train firing rate (z-statistic = 2.20, $p = 0.0275$), average number of spikes in each train (z-statistic = 4.34, $p = 1.4 \times 10^{-5}$), length of the path delineated by trains in centimeters (z-statistic = 1.46, $p = 0.143$), number of 2 cm-by-2 cm spatial bins that the train passed through (z-statistic = 1.14, $p = 0.255$), selectivity (z-statistic = 0.53, $p = 0.595$), sparsity (z-statistic = 0.38, $p = 0.707$), and information (z-statistic = 0.82, $p = 0.411$, MW tests). In addition to confirming that the firing characteristics of control CA3 spike trains showed only minor differences across the two spatial working memory tasks, potential task-related differences between DG and MEC-lesion effects were further minimized by comparing lesion effects to only control data from the same task. **b**, Comparisons of CA3 spike trains from DG-lesion rats (LESION$^{(DG)}$; dark blue) with spike trains from their respective control group (CONTROL$^{(DG)}$; light blue) include the proportion of spikes assigned to trains (z-statistic = 3.99, $p = 6.5 \times 10^{-5}$), in-train firing rate (z-statistic = 1.53, $p = 0.126$), average number of spikes in each train (z-statistic = 0.29, $p = 0.775$), length of the path delineated by trains in centimeters (z-statistic = 2.04, $p = 0.041$), number of 2 cm-by-2 cm spatial bins that the train passed through (z-statistic = 1.31, $p = 0.189$), selectivity (z-statistic = 2.07, $p = 0.039$), sparsity (z-statistic = 1.03, $p = 0.302$), and information (z-statistic = 1.17, $p = 0.243$, MW tests). The control data (light blue) are repeated from **a** for comparison. **c**, Comparisons of CA3 spike trains

Figure S3 continued... from MEC-lesion rats (LESION$^{(MEC)}$; red) with spike trains from their respective control group (CONTROL$^{(MEC)}$; yellow) include the proportion of spikes assigned to trains (z-statistic = 1.82, $p = 0.0685$), in-train firing rate (z-statistic = 2.35, $p = 0.019$), average number of spikes in each train (z-statistic = 2.87, $p = 0.0041$), length of the path delineated by trains in centimeters (z-statistic = 3.07, $p = 0.0021$), number of 2 cm-by-2 cm spatial bins that the train passed through (z-statistic = 1.52, $p = 0.13$), selectivity (z-statistic = 2.20, $p = 0.028$), sparsity (z-statistic = 2.03, $p = 0.043$), and information (z-statistic = 2.09, $p = 0.037$, MW tests). The control data (yellow) are repeated from (a) for comparison. Bars and error bars, mean ± s.e.m., n.s., not significant, * $p < 0.05$, ** $p < 0.01$, *** $p < 0.001$.

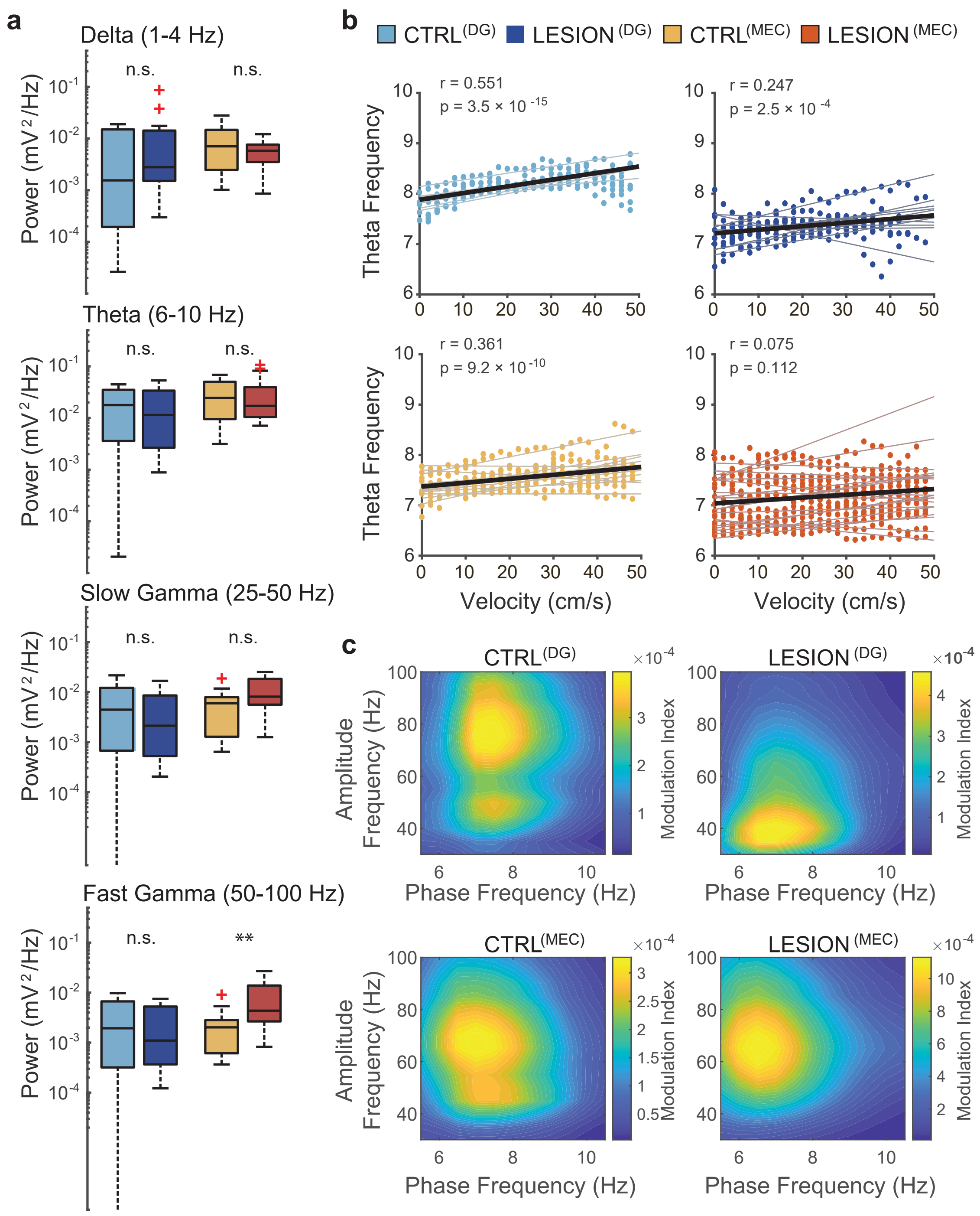

Figure S4. CA3 LFP power did not differ between groups except for an increase in fast gamma in the MEC-lesion group. a, Quantification of CA3 LFP power in different frequency bands. We did not find differences between lesion groups (DG, $n = 11$ sessions; MEC, $n = 20$ sessions) and their matched controls (DG, $n = 7$ sessions; MEC, $n = 13$ sessions) for delta (1-4 Hz: DG, rank sum = 58, $p = 0.48$; MEC, z-statistic = 0.83, $p = 0.41$), theta (6-10 Hz: DG, rank sum = 65, $p = 0.93$; MEC, z-statistic = 0.09, $p = 0.93$), and slow gamma power (25-50 Hz: DG, rank sum = 71, $p = 0.73$; MEC, z-statistic = 1.86, $p = 0.06$, MW tests). However, for fast gamma power (50-100 Hz), we observed an increase in power with MEC, but not with DG lesions (MEC, z-statistic = 2.71, $p = 6.7 \times 10^{-3}$; DG, rank sum = 70, $p = 0.79$, MW tests). Box plots: central line, edges, whiskers and red plus signs indicate median, the 25th/75th percentile, maximum/minimum, and outliers. ** $p < 0.01$. **b**, Theta frequency in

Figure S4 continued... CA3 as a function of running speed. For analyses of theta frequency by running speed, data from all behavior sessions in a group were pooled, and a regression line (black) was fitted to the group data with r and p values for each group reported in each panel. Regression lines (colored) for individual sessions are also shown, and their y-intercepts were used to compare the baseline theta frequency across groups (DG control: n = 7 sessions, 7.92 ± 0.21 Hz; DG lesion: n = 11 sessions, 7.28 ± 0.56 Hz; MEC control: n = 13 sessions, 7.32 ± 0.40 Hz; MEC lesion: n = 20 sessions, 7.07 ± 0.84 Hz, median ± iqr; DG control vs. lesion: rank sum = 103, $p = 2.5 \times 10^{-4}$; MEC control vs. lesion: z-statistic = 2.23, rank sum = 282, p = 0.026; DG vs. MEC control: z-statistic = 2.77, rank sum = 109, $p = 5.6 \times 10^{-3}$; DG vs. MEC lesion: z-statistic = 1.09, rank sum = 203, p = 0.27, MW tests). **c**. Modulation index of theta phase-gamma amplitude modulation for each group. DG lesions did not have an effect on the fast-gamma amplitude (see **a**), but diminished theta-phase modulation of fast gamma amplitude (top, right). The loss of coordination with theta was specific for fast-gamma amplitude, as modulation of slow-gamma amplitude was retained. With MEC lesions, the increase in CA3 fast gamma power (see **a**) was accompanied by a ~3-fold increase in the modulation strength of CA3 fast-gamma amplitude by theta phase (bottom, right), which indicates that theta and gamma oscillations continued to be well coordinated. Our findings suggest qualitatively different effects of DG and MEC inputs on the CA3 LFP with MEC influencing the power of CA3 fast gamma and DG influencing the coordination of CA3 fast-gamma with theta phase.

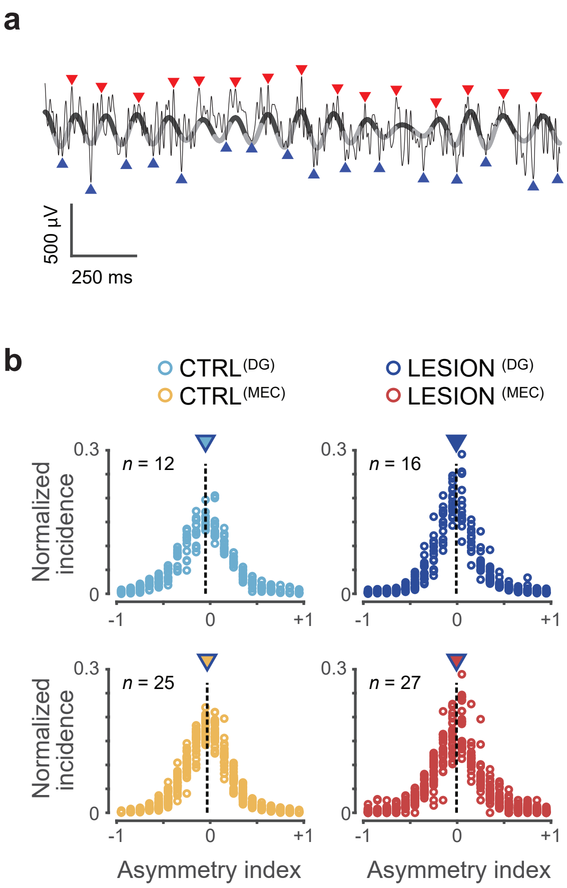

Figure S5. CA3 theta waves showed only minor or no asymmetry in any experimental group. a, Illustration of the method for computing the asymmetry index. To calculate an asymmetry index, we adapted published methods[56] for CA1 LFP, but with minor modifications to account for the higher gamma amplitude in CA3 compared to CA1. We began by bandpass filtering the raw LFP in the theta band (6-10 Hz) and in the broader 1 Hz to 80 Hz band, as in ref. 56. Using the 1-80 Hz bandpass-filtered signal, we then identified the maximum within the first half of the theta cycle (0-180º of the 6-10 Hz filtered signal) and the minimum within the second half of the theta cycle (180º-360º of the 6-10 Hz filtered signal) and marked these extrema by red and blue triangles as peaks and troughs, respectively. The asymmetry index is the ratio of the duration of the ascending wave segment (trough to peak) divided by the duration of the descending wave segments (peak to trough) on a logarithmic scale. Using this scale, zero corresponds to a symmetric wave shape. **b**, To obtain an estimate for wave asymmetry in each recording session, we calculated the mean asymmetry index over all theta cycles in the recording session. The dashed lines (with triangles at the top) are averages over all the recording session means in each group (CTRL$^{(DG)}$: $n = 12$ sessions, -0.0529; LESION$^{(DG)}$: $n = 16$ sessions, -0.0094; CTRL$^{(MEC)}$: $n = 25$ sessions, -0.0332; LESION$^{(MEC)}$: $n = 27$ sessions, -0.0050; CTRL$^{(DG)}$ vs LESION$^{(DG)}$: $p = 0.049$; CTRL$^{(MEC)}$ vs. LESION$^{(MEC)}$: $p = 0.044$; CTRL$^{(DG)}$ vs. CTRL$^{(MEC)}$, $p = 0.17$; MW tests). The number of sessions was higher than for other analyses, because sessions without unit recordings were also included. For plotting the wave asymmetry distribution, we computed the normalized frequency distribution of each session (from -1 to +1 with a bin width of 0.1), and each dot in the plot depicts the value from one bin in each recording session. The means and the distributions indicate that theta wave asymmetry is minor at CA3 recording sites in both control groups, and there was no added asymmetry with either DG and MEC lesions. Given that asymmetry is minor, a Hilbert transform is appropriate for theta phase estimates and corrections for asymmetric wave shape are not needed when using CA3 LFP recordings.

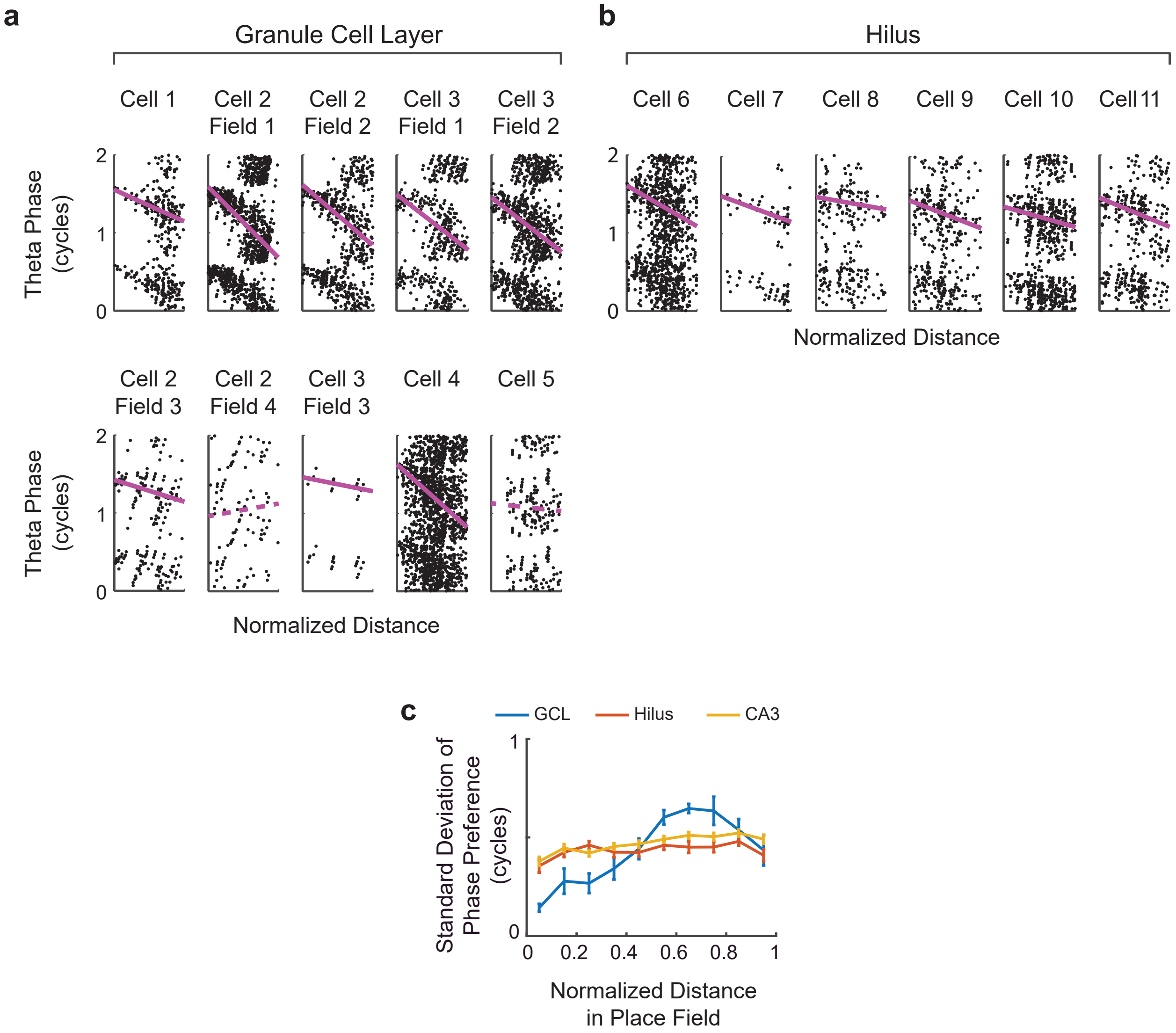

Figure S6. Neurons recorded in the dentate granule cell layer showed the lowest spike phase variability at the entry into place fields. a, Place fields were defined as the area within the 20% contour of the place maps, and normalized distances along the path from the entry to the exit were calculated. Phase-distance plots of all neurons that were recorded on tetrodes that were confirmed to terminate in the granule cell layer (GCL, $n = 5$ cells, 10 place fields from 3 rats). **b**, Six phase-distance plots from neurons that were recorded from tetrodes that were confirmed to terminate in the dentate gyrus but in the hilar region or the subgranular zone rather than the granule cell layer (Hilus, total sample, $n = 13$ neurons, 34 place fields from 4 rats). **c**, Quantification of theta phase variability (circular standard deviation) as mean ± s.e.m at various distances through the place field. These plots show that, compared to hilar and CA3 cells, putative granule cells tend to fire at a consistent phase at the entry into the place field. Granule cells could therefore provide a reliable input that controls the onset phase of CA3 pyramidal cell spiking.

Figure S7

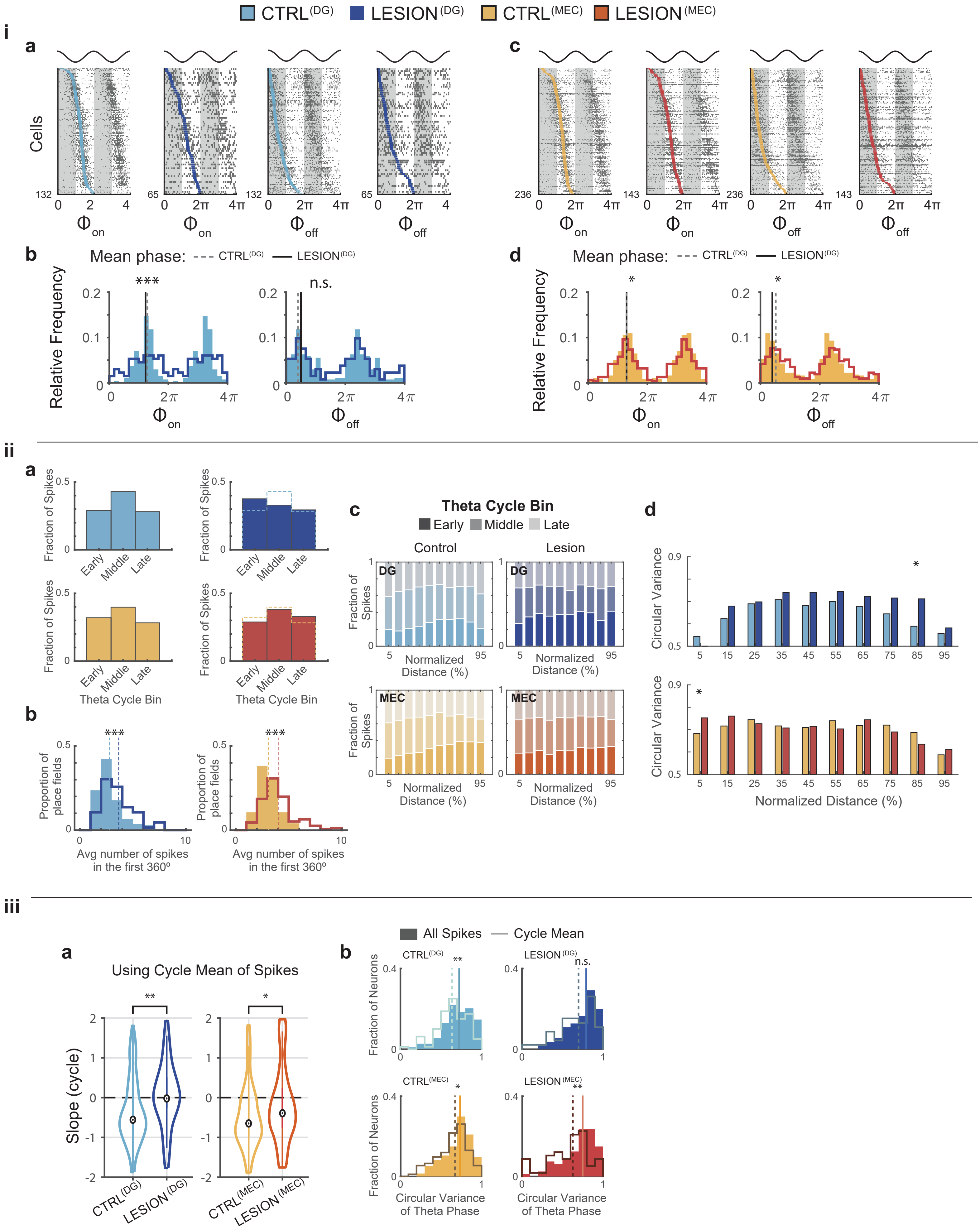

CTRL(DG) LESION(DG) CTRL(MEC) LESION(MEC)
i
a
c
Cells
Φon
Φoff
b
Mean phase: CTRL(DG) LESION(DG)
Relative Frequency
***
n.s.
d
*
ii
a
Fraction of Spikes
Early Middle Late
Theta Cycle Bin
b
Proportion of place fields
Avg number of spikes in the first 360º
c
Theta Cycle Bin
Early Middle Late
Control
Lesion
DG
MEC
Fraction of Spikes
Normalized Distance (%)
d
Circular Variance
Normalized Distance (%)
iii
a
Using Cycle Mean of Spikes
Slope (cycle)
CTRL(DG) LESION(DG) CTRL(MEC) LESION(MEC)
**
*
b
All Spikes
Cycle Mean
CTRL(DG)
LESION(DG)
CTRL(MEC)
LESION(MEC)
Fraction of Neurons
Circular Variance of Theta Phase

Figure S7. Analyses corresponding to Figs. 3, 4 and 5, but by selecting spikes in place fields instead of spike trains. Place field-based analyses support the same conclusions as train-based analyses. The place field of the neuron was defined as the area within the 20% contour of the place maps. **i**, reproduction of Fig. 3 using identified CA3 place fields rather than spike trains as the basis for analysis. **a**, Onset and offset phases of CA3 spikes in control ($CTRL^{(DG)}$) and DG-lesion rats ($LESION^{(DG)}$). **b**, The cells' mean onset and offset phases were compared between control (light blue bars) and DG-lesion rats (dark blue line). The distribution of onset phases differed between control and DG-lesion rats (n = 132 $CTRL^{(DG)}$ place fields and n = 65 $LESION^{(DG)}$ place fields, mean onset phase: 221.5º vs. 233.2º, $\chi 2 = 29.3$, $p = 4.3 \times 10^{-7}$, circular MANOVA) with a higher concentration in the control compared to the lesion group (phase concentration: $\kappa = 1.87$ vs. $\kappa = 0.55$, U = 20.7, $p = 5.3 \times 10^{-6}$, concentration test). Offset phases were not changed by the lesion (90.5º vs. 72.6º, $\chi 2 = 4.0$, $p = 0.13$, circular MANOVA; phase concentration: $\kappa = 1.14$ vs. $\kappa = 0.82$, U = 1.5, $p = 0.22$, concentration test). **c** and **d**, as (a and b), but for CA3 cells from MEC-lesion rats (red line) and their corresponding controls (yellow bars). Onset phase values peaked in the ascending phase of the theta cycle, with their distribution only slightly altered by the lesion (n = 236 $CTRL^{(MEC)}$ and 143 $LESION^{(MEC)}$ fields, mean onset phase: 233.8º vs. 230.4º, $\chi 2 = 8.3$, $p = 0.016$, circular MANOVA; phase concentration: $\kappa = 1.61$ vs. $\kappa = 1.05$, U = 7.6, $p = 0.0059$, concentration test). Similarly, the distribution of offset phases was slightly different between cells of control and MEC-lesion rats (72.5º vs. 93.4º, $\chi 2 = 6.2$, $p = 0.046$, circular MANOVA; concentration: $\kappa = 1.11$ vs. $\kappa = 0.95$, U = 0.80, $p = 0.37$, concentration test). **ii**, reproduction of Fig. 4, but by using identified CA3 place fields rather than spike trains. Panels are presented as in Fig. 4.
a, Compared to control data, CA3 spike phases in the theta cycle were markedly shifted with reduced DG granule cell input (mean theta phase: 166.1º vs. 86.9º, $\chi 2 = 39.4$, $p = 2.7 \times 10^{-9}$, circular MANOVA test). In contrast, there was no significant difference in CA3 spike phases between MEC control and lesion data (mean theta phase: 160.6º vs. 177.1º, $\chi 2 = 3.9$, $p = 0.14$, circular MANOVA test). **b**, Average number of spikes over a 360º-cycle from the first spike within the place field. In trains from cells of DG-lesion rats, the median number of initial-cycle spikes (indicated by dashed lines) was 2.80, 3.67, 3.08, and 4.07 for $CTRL^{(DG)}$, $LESION^{(DG)}$, $CTRL^{(MEC)}$, and $LESION^{(MEC)}$ ($CTRL^{(DG)}$ vs. $LESION^{(DG)}$, z-statistic = -3.8, $p = 1.7 \times 10^{-4}$; $CTRL^{(MEC)}$ vs. $LESION^{(MEC)}$, z-statistic = -6.1, $p = 1.4 \times 10^{-9}$, MW tests). **c**, Fraction of CA3 spikes at early, middle, and late phases of the theta cycle as a function of normalized position (%) within the linearized place field. At the onset of control trains, a low proportion of spikes is typically observed at early phases, but this proportion increased with DG lesions. **d**, Circular variance of spikes as a function of normalized position (in % of field extent) within the linearized place field. For DG lesions, effects were not as pronounced with field-based than with spike train-based analysis. **iii**, reproduction of Fig. 5c and d, but by using identified CA3 place fields rather than spike trains. Panels are presented as in Fig. 5c and d.
a, Distribution (violin plot) of circular-linear regression slopes calculated from the cycle means. Using the regression slopes from cycle means there was a difference between cells from control and MEC-lesion rats (z-statistic = -2.29, $p = 0.022$, MW test), and from control and the DG-lesion rats (z-statistic = -3.26, $p = 0.0011$, MW test). **b**, Circular variance of theta phase with either all spikes (filled bars) or with each cycle's spike mean (solid lines). Vertical lines indicate median of the color-matched histogram. The circular variance decreased after replacing each cycle's spikes with their mean, except for CA3 cells of DG-lesion rats ($CTRL^{(DG)}$: z-statistic = 2.95, $p = 0.0032$; $LESION^{(DG)}$: z-statistic = 1.73, $p = 0.084$; $CTRL^{(MEC)}$: z-statistic = 2.38, $p = 0.017$; $LESION^{(MEC)}$: z-statistic = 3.15, $p = 0.0016$; MW tests). Violin plots: Outline, distribution; error bars: 1.5 times the interquartile interval above the third and below the first quartile. n.s., not significant, * $p < 0.05$, ** $p < 0.01$, *** $p < 0.001$.

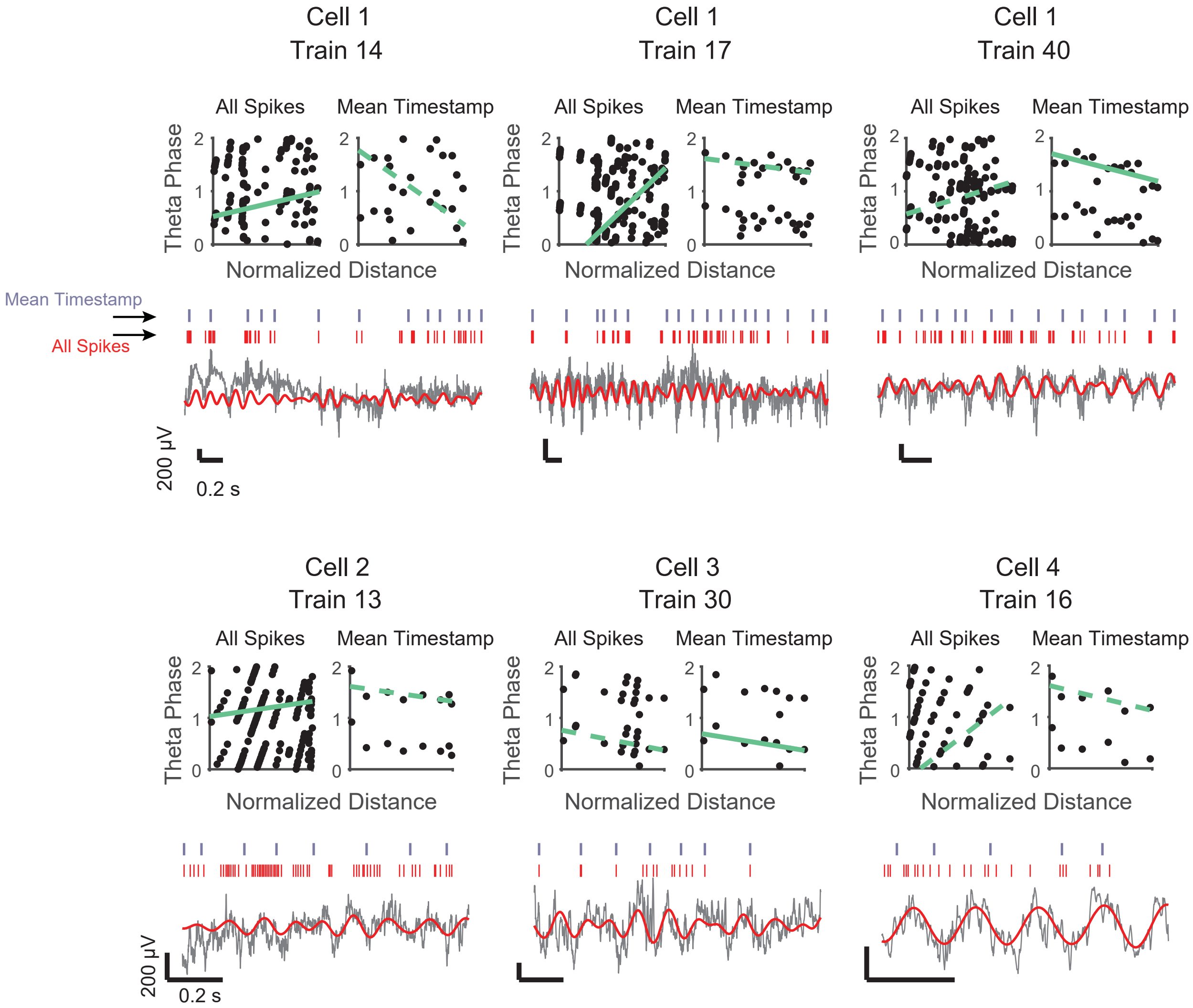

Figure S8. Replacing each theta cycle's spike phases with the mean phase within the cycle rescued CA3 phase precession in cells from the MEC-lesion, but not from the DG-lesion group. Examples of CA3 spike trains from the MEC lesion group. Replacing each theta cycle's spike phases with the mean phase results in a negative slope when a positive slope was detected with the unprocessed spike phases. Below the phase-distance plots, the spike train (red ticks) and the mean timestamps of each cycle's spikes (blue ticks) are displayed along with the simultaneously recorded LFP (gray, unfiltered, red, 6-10 Hz filtered). Solid green lines, statistically significant single-train slopes, stippled green lines, non-significant single-train slopes.

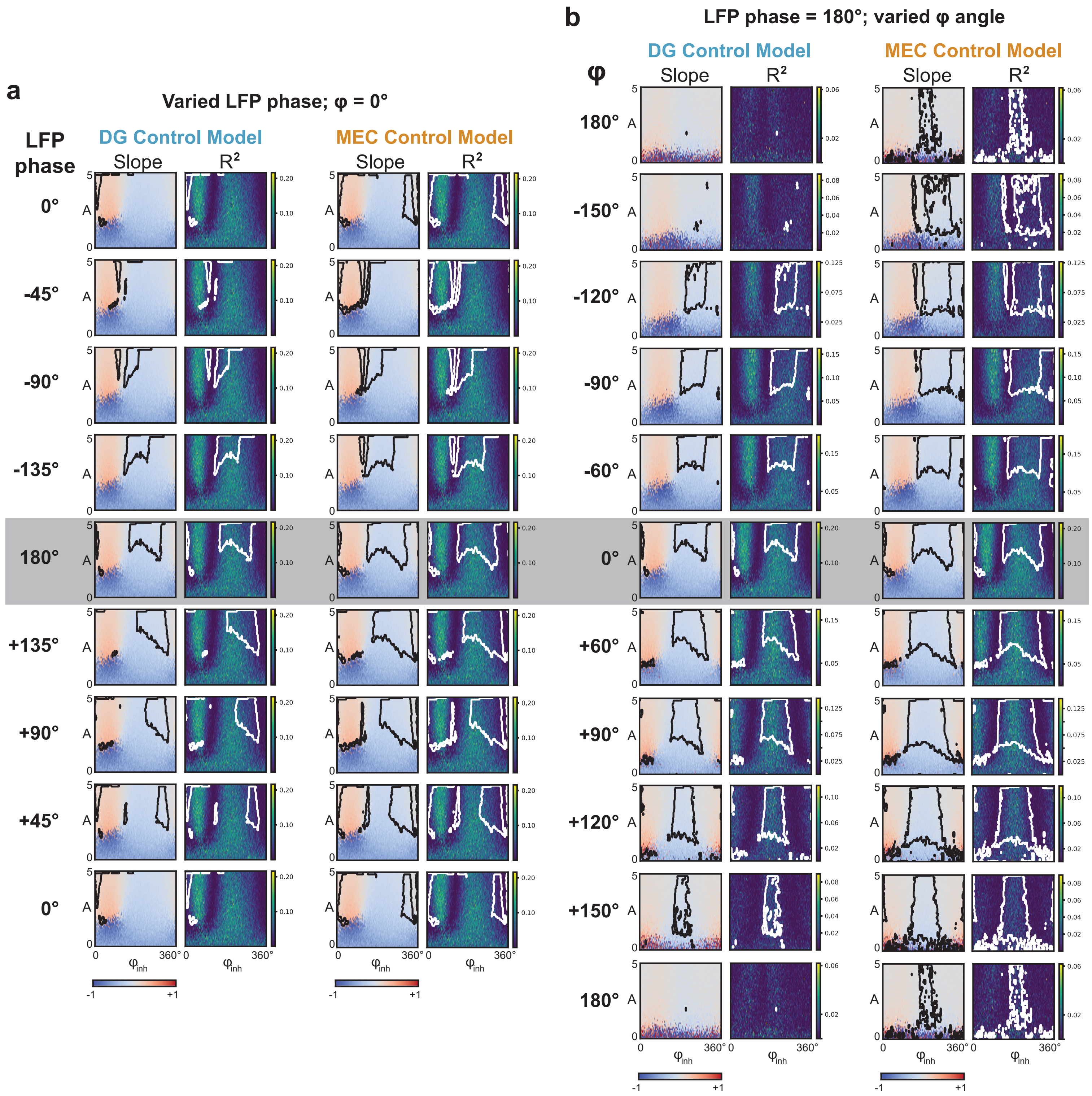

Figure S9. The model best matched empirical data when the maximal CA3 firing was aligned with the time of minimum inhibition and when phase shifts between the two excitatory inputs were ≤90°. a, The theta phase difference (i.e., LFP phase) between empirical data and the model data was varied in 45° steps while the phase angle of the two excitatory inputs was aligned (ψ = 0º). At an LFP phase angle of 180°, the maximal CA3 firing rate occurred at the phase of minimum inhibition in the model. This alignment between the empirical LFP and the model inhibitory phase resulted in a broad range of A and φ_{inh} parameter combinations to reproduce empirical control data and was therefore selected for all further analyses of the correspondence between empirical data and the model. **b**, The phase angle of the two excitatory inputs (ψ) was varied in 30º steps while the theta phase difference (i.e., LFP phase) was fixed at 180° degrees. Phase differences between the two excitatory inputs up to ±90º did not result in major variation in the allowable A-φ_{inh} parameter space that matched control data. The color code of all figure panels is as in Fig. 7d. The panels that are highlighted by the gray background correspond to the parameters that are used in Fig. 7.

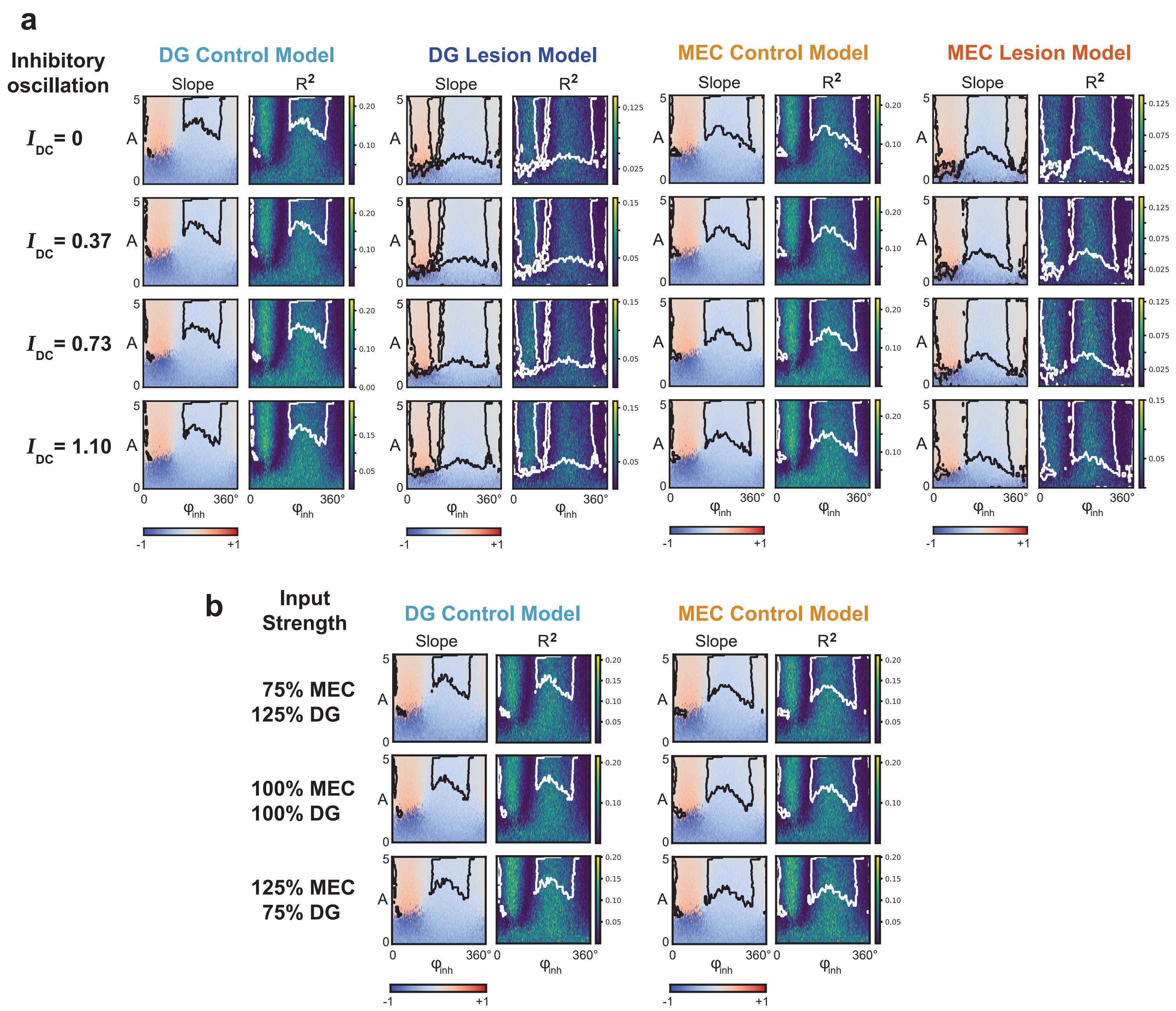

Figure S10. Baseline inhibition level (I_{DC}) and variation in the balance of excitatory amplitudes do not qualitatively alter the model A-φ_{inh} parameter space that fits to empirical data. a, The inhibitory steady-state level (I_{DC}) was varied while other model parameters remained fixed (LFP phase = 180°, ψ = 0º). Four levels of inhibition (I_{DC} = 0, 0.37, 0.73 or 1.10) are shown, along with the A-φ_{inh} parameter space of the control and DG-lesion and the control and MEC-lesion models. For each of the model parameters, the match to empirical data is depicted by outlines (black or white). It is evident that even considerable variation in I_{DC} did not result in major changes in either the A-φ_{inh} parameter space or the model fit to empirical data. The color code of all figure panels is as in Fig. 7d, and the top row (I_{DC} = 0) corresponds to the model used in Fig. 7. **b**, The relative strength of the two excitatory inputs (DG and MEC input) to the model was varied while other model parameters remained fixed (LFP phase = 180° degrees, ψ = 0º, I_{DC} = 0). The total level of excitatory input was held constant so that the two inputs added to 200%. Substantial variation in the relative strength of each of the two excitatory inputs did not result in major changes in either the A-φ_{inh} parameter space or the model fit to empirical data. The color code of all figure panels is as in Fig. 7d, and the middle row (100% MEC, 100% DG) corresponds to the model used in Fig. 7.

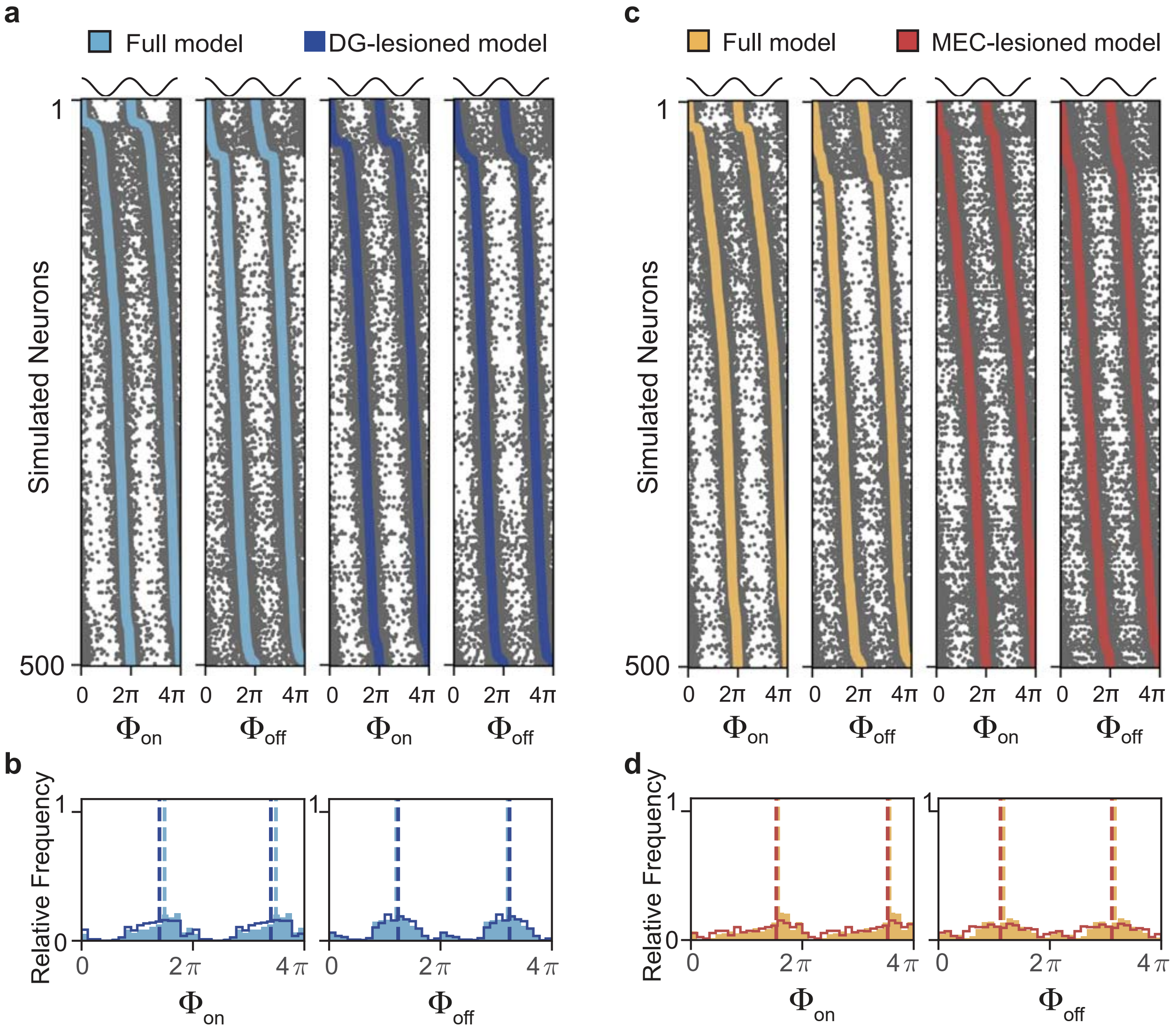

Figure S11. Reproduction of spiking data using the computational model. a, Onset and offset phases of spike trains from simulated CA3 neurons using a control instance of the model (full model) and an instance with the DG excitatory input set to zero (DG-lesion model). The figure panels are depicted as in Fig. 3, with gray ticks on each line indicating the onset (Φon) or offset (Φoff) phases of the spike trains of a simulated cell. A total of 500 cells were simulated. Each row's median is marked by a light blue (full model) or dark blue tick (DG-lesion model). Data are repeated from 2π to 4π for clarity. Different cells used different A-φ_{inh} parameter pairs randomly sampled from the allowable parameter space (90% quantile centered on the median). **b**, Histogram of onset (left) and offset (right) phases of simulated spikes for the full model (light blue) and the DG-lesion model (dark blue). The DG-lesion model generates broader and earlier onset phases for CA3 spike trains. Dashed vertical lines, circular means of color-matched histogram. **c** and **d**, as in (a and b), but with using a control instance of the model (full model) and an instance with the MEC excitatory input set to zero (MEC-lesion model). The full model is shown in yellow and the MEC-lesion model in red. The MEC-lesion model generates onset phases for CA3 spike trains that match the full model.

Table S1. Summary of rats used in this study.

RAT ID	EXPERIMENT	EXPERIMENTAL CONDITION	# QUALIFYING CA3 UNITS	# QUALIFYING DG UNITS	# SESSIONS
**122**	DG	CTRL	N/A	5	1
**147**	DG	CTRL	N/A	6	1
**194**	DG	CTRL	N/A	4	1
**600**	DG	CTRL	50	N/A	2
**601**	DG	CTRL	19	2	2
**632**	DG	CTRL	7	1	2
**650**	DG	CTRL	8	N/A	1
		**CTRL$^{(DG)}$ TOTAL**	84	18	10
**599**	DG	LESION	4	N/A	1
**633**	DG	LESION	5	N/A	2
**648**	DG	LESION	11	N/A	2
**649**	DG	LESION	4	N/A	2
**656**	DG	LESION	2	N/A	1
**669**	DG	LESION	20	N/A	2
**672**	DG	LESION	7	N/A	3
**675**	DG	LESION	7	N/A	1
**684**	DG	LESION	8	N/A	2
		**LESION$^{(DG)}$ TOTAL**	68	N/A	16
**3661**	MEC	CTRL	23	N/A	5
**3839**	MEC	CTRL	11	N/A	3
**3840**	MEC	CTRL	2	N/A	1
**3906**	MEC	CTRL	13	N/A	2
**3931**	MEC	CTRL	4	N/A	1
**3958**	MEC	CTRL	39	N/A	3
**3959**	MEC	CTRL	9	N/A	3
		**CTRL$^{(MEC)}$ TOTAL**	101	N/A	18
**3656**	MEC	LESION	5	N/A	1
**3754**	MEC	LESION	17	N/A	5
**3756**	MEC	LESION	10	N/A	2
**3837**	MEC	LESION	6	N/A	2
**3903**	MEC	LESION	9	N/A	2
**3928**	MEC	LESION	29	N/A	3
**3978**	MEC	LESION	48	N/A	3
**3979**	MEC	LESION	34	N/A	2
		**LESION$^{(MEC)}$ TOTAL**	158	N/A	20

Table S2. Statistics from Mann-Whitney tests performed for Fig. 4d.

CTRL$^{(DG)}$ vs LESION$^{(DG)}$:

BIN	1	2	3	4	5	6	7	8	9	10
**Z-STATISTIC**	-2.79	-2.78	-2.83	-2.75	-2.98	-0.79	-0.19	-0.42	-1.36	-1.02
**U**	4863.5	3906	4369.5	4113	4695.5	4916.5	5374.5	5353.5	4825.5	5006
**P-VALUE**	0.0051	0.0053	0.0045	0.0058	0.0028	0.42	0.84	0.67	0.17	0.3
**HOLM-BONFERRONI CORRECTION**	*	*	*	*	*	n.s.	n.s.	n.s.	n.s.	n.s.

CTRL$^{(MEC)}$ vs LESION$^{(MEC)}$:

BIN	1	2	3	4	5	6	7	8	9	10
**Z-STATISTIC**	-2.35	-0.5	-1.54	-1.01	-0.73	0.29	-0.7	-1.62	-0.88	-0.85
**U**	11024	10570	10708	10138	11089	11773	11314	10800	10446	11557
**P-VALUE**	0.0183	0.6145	0.1214	0.3105	0.4628	0.7677	0.4791	0.1052	0.3737	0.39
**HOLM-BONFERRONI CORRECTION**	n.s.	n.s.	n.s.	n.s.	n.s.	n.s.	n.s.	n.s.	n.s.	n.s.

* significant at alpha = 0.05, n.s., not significant at alpha = 0.05

References

93. Ainge, J.A., van der Meer, M. A., Langston, R. F. & Wood, E. R. Exploring the role of context-dependent hippocampal activity in spatial alternation behavior. *Hippocampus*. **17**, 988-1002 (2007).
